# Supplementary material for: Content-rich biological network constructed by mining PubMed abstracts
Source: BMC Bioinformatics. 2004 Oct 8;5:147. doi: 10.1186/1471-2105-5-147 (PMC528731; doi:10.1186/1471-2105-5-147)
Supplement: Additional File 2 — The original results of the above study (non-essential files are deleted to keep the file size under the limit set by BMC bioinformatics). [file 1471-2105-5-147-S2.bz2 › chilibotAdditionalFile2/dip05/414ID10504300E10080/html/BMP6_ACVR1.html]

 


 **BMP6** and **ACVR1** 
  
Found 7 abstracts in PubMed, retrieved 05.  
 

 What does Google say? 
 PDF only 
| .edu only 

---

**Interactive relationship** (e.g. stimulation, inhibition, etc)

**Neutral relationship**- ... BMP 6  [ **BMP6** ]  and BMP 7 preferentially bind to ALK 2  [ **ACVR1** ] .  Ref: 11282024 J Cell Sci, 2001
